# A rare cause of MINOCA: embolism of a thrombus arising from accessory mitral valve tissue

**DOI:** 10.1093/ehjcr/ytaf222

**Published:** 2025-05-06

**Authors:** En Ze Chan, Giap Swee Kang, Amelia Xin Chun Goh

**Affiliations:** Department of Cardiology, National University Heart Centre, National University Health System, 1E Kent Ridge Road, Singapore 119074, Singapore; Sarawak Heart Center, Kuching - Samarahan Expressway, 94300 Kota Samarahan, Sarawak, Malaysia; Faculty of Medicine and Health Sciences, Universiti Malaysia Sarawak, Jln Datuk Mohammad Musa, 94300 Kota Samarahan, Sarawak, Malaysia; Yong Loo Lin School of Medicine, National University of Singapore, 10 Medical Dr, Singapore 117597, Singapore; Department of Cardiac, Thoracic and Vascular Surgery, National University Heart Centre, National University Health System, 1E Kent Ridge Road, Singapore 119074, Singapore; Department of Pathology, National University Hospital, National University Health System, 1E Kent Ridge Road, Singapore 119074, Singapore

**Figure:**
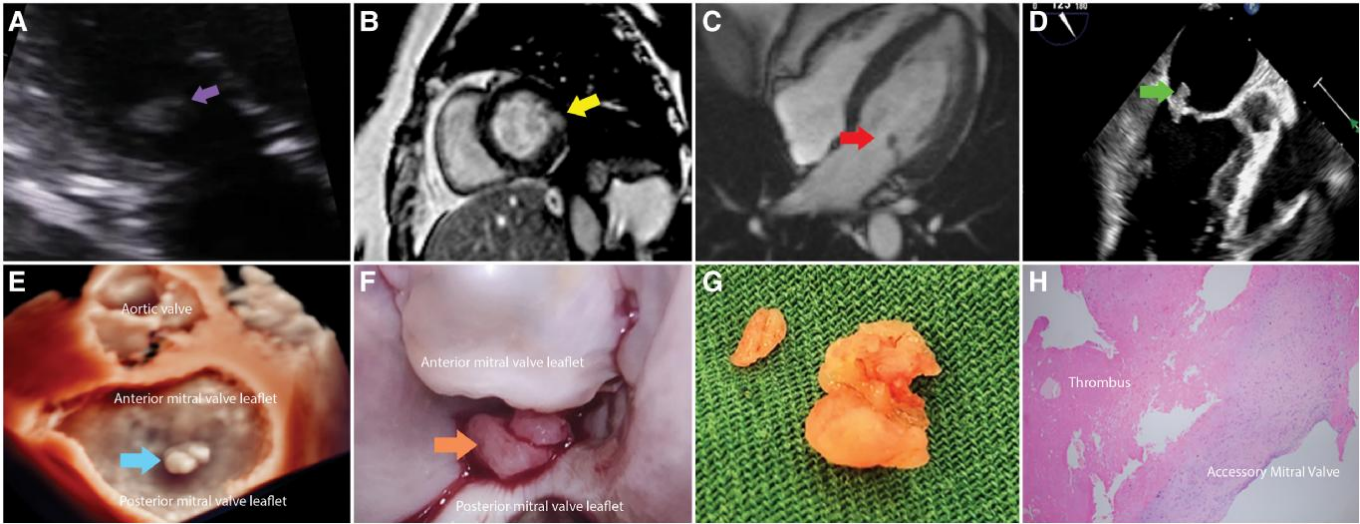


A 51-year-old lady with underlying hypertension presented with angina. Electrocardiogram showed no ischaemic changes, but the highly sensitive troponin I was 203.3 ng/L (reference range ≤ 26.2 ng/L). Transthoracic echocardiogram demonstrated a left ventricular ejection fraction of 65% with no regional wall motion abnormality but suspicion of posterior mitral valve (MV) leaflet mass with trivial mitral regurgitation with no evidence of valve stenosis (*Panel A*; Supplementary material online, *Video S1*). Coronary angiogram demonstrated 20% stenosis at the distal left anterior descending artery. Therefore, a diagnosis of myocardial infarction with non-obstructive coronary arteries (MINOCA) was made and was treated with dual antiplatelet therapy. The cardiovascular magnetic resonance (CMR) performed 3 weeks later demonstrated focal near transmural late gadolinium enhancement of the basal anterolateral segment, confirming the diagnosis of myocardial infarction (*Panel B*). Incidentally, a mobile mass on cine images was attached to the posterior MV leaflet (*Panel C*). A transesophageal echocardiogram demonstrated a mobile bilobed elongated mass measuring 17 × 6 mm arising from the tip of the P2 leaflet of the MV on the atrial aspect with trivial mitral regurgitation (*Panels D* and *E*; Supplementary material online, *Videos S2* and *S3*). C-reactive protein and three sets of blood cultures were negative, excluding infective endocarditis.

She underwent minimally invasive excision of MV mass. Intraoperatively, the bilobed mass (2 × 1 cm) was attached to the tip of the P2 MV leaflet (*Panel F*) and was excised en bloc, preserving the MV. The mass appeared to have an irregular papillary surface (*Panel G*). Histopathology demonstrated normal accessory MV tissue with attached white thrombus (*Panel H*). The patient recovered well, and the echocardiogram showed a good MV function with trivial mitral regurgitation at follow-up. This case demonstrated the thrombus embolization arising from accessory MV tissue causing MINOCA.

## Supplementary material

Supplementary material is available at *European Heart Journal – Case Reports* online.

**Consent:** The patient provided written informed consent for data collection and publication. This manuscript does not provide personal identifying information.

**Funding:** None declared.

## Supplementary Material

ytaf222_Supplementary_Data

## Data Availability

The data underlying this article will be shared on reasonable request to the corresponding author.

